# Response to CATCH-IT Report by Cameron Norman: Evaluation of an Internet-Based Smoking Cessation Program: Lessons Learned From a Pilot Study

**DOI:** 10.2196/jmir.6.4.e48

**Published:** 2004-12-31

**Authors:** Edward G Feil

**Affiliations:** ^2^Institute on Violence and Destructive BehaviorCollege of EducationUniversity of OregonEugene ORUSA; ^1^Oregon Research InstituteEugene ORUSA

Cameron Norman's critique [1] of our brief report in *Nicotine & Tobacco Research* [2] provides a good summary of its contents. The author makes several legitimate suggestions, but perhaps does not stress enough the core issues of our original study. These issues are

1. comparatively equal effectiveness of Internet-based health interventions and intensive clinical treatment;
2. the methodological problem of assignment to a control condition;
3. high attrition at follow-up assessments.

As one of the authors of the original report I take this opportunity to respond to some of the design criticisms noted in the CATCH-IT report.

Our small pilot study was presented as a brief report; it was therefore limited in scope and not as detailed as a full manuscript. Several items such as randomization, mean time to follow-up, and measures collected (ie, other smoking strategies and cessation self-efficacy, and social support) could not be described in detail in a brief report.

Our report [2] noted that while

these results provide reason for further evaluations. ... However, given the lack of a control condition, we cannot conclude that quitting was a function of our Web site rather than other factors. Determining the relative contribution of a specific Web site presents difficult challenges, given that typical Internet users appear to sample various sites.

Therefore, an alternative treatment is only a click away, especially for a person seeking out a website for help in smoking cessation. We surmised that given the nature of the Internet, any assignment to a control condition would be futile. Additionally, the welcoming anonymity of the Internet combined with the transitory nature of email addresses makes follow-up difficult. Unless this methodological problem can be addressed by Internet health researchers, a true randomized trial on the open Internet is untenable and definitive results will remain elusive [3].

The CATCH-IT author states that no reference was made to a specific theoretical model for the intervention, while noting that our brief report did cite Lichtenstein and Glasgow [4]. This paper notes that behavioral intervention with relapse prevention is the type of treatment most used in intensive clinical settings, and is the most effective.  Our pilot study attempted to use the most intensive clinical and empirically-proven approach and adapt it for delivery over the Internet.

The amount of intervention exposure (dose) was assessed and measured for each participant through the use of a fairly standard tracking system for an Internet-based intervention, namely, the number of log-ins. Our brief report [2] did state that

The Web site recorded 24 252 logins (i.e., instances when a participant used a username and password to gain access to the Web site), with an average of 108 logins per day. … Most activity occurred immediately after completion of baseline assessment and on weekdays. … Considerable variation in the number of logins was noted, with 10% of the participants accounting for 79% of logins. … While the gender differences and participation rates might indicate trends, these results were not statistically significant.

The CATCH-IT author was concerned with the lack of biochemical verification of self-reported abstinence in the context of a “remotely-delivered intervention.” [1] According to the Society for Research on Nicotine and Tobacco Subcommittee on Biochemical Verification [5], the decision to use biochemical validation of tobacco use depends on three issues: demand characteristics, type of study, and type of population. Biochemical verification is recommended in randomized clinical trials of intensive interventions where repeated contacts between research or intervention personnel and subjects might elicit relatively high demand characteristics. The authors review several recent large-scale studies and conclude that biochemical verification is not warranted in population-based interventions with limited face-to-face contact. As to the impact of inaccurate self-report, the Subcommittee states that while it is likely that self-report will inflate quit rates, the magnitude of such inflations is small. 

Overall, this particular CATCH-IT report (and - hopefully - future CATCH-IT reports in this new series in the *Journal of Medical Internet Research*) lays the groundwork for a discussion of the important issues germane to Internet-based health interventions. The need for rigorous evaluation of randomized controlled trials is imperative as the number of Internet-based health interventions promoted to the public increases. Such emphasis on high standards will help to prevent dissemination of unproven interventions and to provide effective programs to Internet and World Wide Web users.

## References

[1] Norman C (2004). CATCH-IT Report: Evaluation of an Internet-based smoking cessation program: lessons learned from a pilot study. J Med Internet Res.

[2] Feil Edward G, Noell John, Lichtenstein Ed, Boles Shawn M, Mckay H Garth (2003). Evaluation of an Internet-based smoking cessation program: lessons learned from a pilot study. Nicotine Tob Res.

[3] Eysenbach Gunther (2002). Issues in evaluating health websites in an Internet-based randomized controlled trial. J Med Internet Res.

[4] Lichtenstein E, Glasgow R E (1992). Smoking cessation: what have we learned over the past decade?. J Consult Clin Psychol.

[5] SRNT Subcommittee on Biochemical Verification (2002). Biochemical verification of tobacco use and cessation. Nicotine Tob Res.

